# Medicinal Plants Based Products Tested on Pathogens Isolated from Mastitis Milk

**DOI:** 10.3390/molecules22091473

**Published:** 2017-09-04

**Authors:** Claudia Pașca, Liviu Mărghitaș, Daniel Dezmirean, Otilia Bobiș, Victorița Bonta, Flore Chirilă, Ioana Matei, Nicodim Fiț

**Affiliations:** 1 Department of Apiculture and Sericiculture, University of Agricultural Sciences and Veterinary Medicine, Cluj-Napoca 400372, Romania; claudia.pasca@usamvcluj.ro (C.P.); lmarghitas@usamvcluj.ro (L.M.); ddezmirean@usamvcluj.ro (D.D.); 2 Life Science Institute “King Michael I of Romania”, University of Agricultural Sciences and Veterinary Medicine, Cluj-Napoca 400372, Romania; obobis@usamvcluj.ro (O.B.); victorita.bonta@usamvcluj.ro (V.B.); 3 Department of Microbiology (Veterinary Medicine), University of Agricultural Sciences and Veterinary Medicine, Cluj-Napoca 400372, Romania; flore.chirila@usamvcluj.ro (F.C.); nfit@usamvcluj.ro (N.F.)

**Keywords:** bovine mastitis, antibiotic resistance, plant alcoholic extract, plant derived products

## Abstract

Bovine mastitis a major disease that is commonly associated with bacterial infection. The common treatment is with antibiotics administered intramammary into infected quarters of the udder. The excessive use of antibiotics leads to multidrug resistance and associated risks for human health. In this context, the search for alternative drugs based on plants has become a priority in livestock medicine. These products have a low manufacturing cost and no reports of antimicrobial resistance to these have been documented. In this context, the main objective of this study was to determine the antimicrobial effect of extracts and products of several indigenous, or acclimatized plants on pathogens isolated from bovine mastitis. A total of eleven plant alcoholic extracts and eight plant-derived products were tested against 32 microorganisms isolated from milk. The obtained results have shown an inhibition of bacterial growth for all tested plants, with better results for *Evernia prunastri*, *Artemisia absinthium,* and *Lavandula angustifolia*. Moreover, *E. prunastri*, *Populus nigra*, and *L. angustifolia* presented small averages of minimum inhibitory and bactericidal concentrations. Among the plant-derived products, three out of eight have shown a strong anti-microbial effect comparable with the effect of florfenicol and enrofloxacin, and better than individual plant extracts possibly due to synergism. These results suggest an important anti-microbial effect of these products on pathogens isolated from bovine mastitis with a possible applicability in this disease.

## 1. Introduction

Bovine Mastitis (BM) is a major disease affecting dairy cattle worldwide, results from the inflammation of the mammary gland, and it is considered to be the most frequent and costly disease of dairy cattle all over the world [1]. It is commonly associated with bacterial infection, being influenced by many and multiple factors related to management, housing, and milking [1]. Depending on the severity of the inflammation, mastitis can be classified into sub-clinical, clinical, and chronic forms, and its degree is dependent on the etiology and on the age, breed, immunological health, and lactation state of the animal [2]. In contrast with the clinical form, subclinical mastitis shows no visible changes in milk or udder but decrease milk production, and bacteria are presented in the secretion [2]. Financial losses due to mastitis occur for both subclinical and clinical stages of the disease and include costs of treatment, discharged milk, transient reductions in milk yield, premature culling, and death. Worldwide, mastitis is associated with economic losses of $35 billion annually [3]. The most common treatment is with antibiotics administered intramammary into the infected quarters of the udder and intramuscular injection [4]. The repeated use of antibiotics to treat BM for a long period may cause multidrug resistance in causative organisms, which will require increased doses of antibiotics leading to the accumulation of large amount of antibiotics in milk and its products. Both the emergence of multidrug resistance strains and the presence of antibiotics residue in food product are important health risks for humans [5].

In this context of increasing multidrug resistance and the demand for organic products, the search of alternative drugs based on the pharmacological and phytochemical properties of plants became a priority in livestock health research [6]. Moreover, the high costs of the synthetic drugs and their various side effects justify the search for alternative products from plants. These products, in addition to a low manufacturing cost, may have other benefits as observed for *Atremisia annua* used as an alternative coccidiostatic, which also increase weight gain, egg production rate, size, and the intensity of egg yolk [7]. In addition, no reports of antimicrobial resistance to these phytochemicals have been documented, probably due to their multiple mechanisms of action that potentially prevent the selection of resistant strains of bacteria [8]. 

There are an increasing number of published studies in the field of antimicrobial therapy using natural products [6,9], including studies on the antimicrobial effect of plant products on pathogens isolated from BM [10,11,12]. However, the majority of these studies are focused on plants which have a natural distribution, specific to certain ecoregions or geographical areas, such as *Centella asiatica* [13], *Punica granatum* [14,15], or *Tridax procumbens* [16]. Despite the encouraging results of these studies, more studies including indigenous or acclimatized plants are required to cover distinct geographical areas in order to have a great availability and a low manufacturing cost for these products.

Romania is a country located in south-eastern Central Europe, on the Lower Danube, in the north of the Balkan Peninsula, and on the northwestern coast of the Black Sea. The climate of Romania is generally temperate-continental, but the country’s relief influences it locally [17]. Romania has five biogeographic regions: continental, steppic, alpine, pannonian, and pontic, resulting in a rich biodiversity [18].

Previous studies have evaluated the antimicrobial effect of several Romanian indigenous medicinal plants on different collection strains of pathogens [17,19,20,21,22,23,24,25,26,27,28,29]. However, only a few studies are focused on the effect on pathogens isolated from animals [30] or humans [31], which may present a higher multidrug resistance. 

In this context, the main objective of this study was to determine the antimicrobial effect of the extracts from indigenous or acclimatized plants on pathogens isolated from clinical and sub-clinical BM, and develop different products for using as alternative natural treatment in BM.

## 2. Results

### 2.1. Determination of Phenolic Compounds Content

The results of the total phenolic content and flavones of plant alcoholic extracts are given in Table 1. The total phenolic content was expressed as gallic acid equivalents (mg·GAE/g plant material) and the calculation of flavones content was performed by using a standard curve of quercetin and presented as quercetin equivalents (mg·QE/g plant material). The total polyphenol content of the studied plant extracts ranges between 2.4–79.2 mg/g DW. The highest amount of phenolic compounds (79.2 ± 1.36 mg·GAE/g DW) was identified at *A. absinthium*. The lowest concentration of total polyphenols is recorded in alcoholic extracts: *Populus nigra* (2.40.45 mg·GAE/g DW) and *Althaea officinalis* (3.3 ± 0.61 mg·GAE/g DW).

In the case of flavones, the order is slightly changed. Thus, the flavones are found in the highest concentration in alcoholic extract of *Mentha pulegium* (150.6 ± 1.51 mg·QE/g DW), followed by *Agastache foeniculum* (89.0 ± 1.01 mg·QE/g DW) (Table 1).

### 2.2. Antioxidant Activity Assays

The DPPH method (2,2-diphenyl-1-picryl-hydrazyl) is one of the most common methods of in vitro determination of antioxidant capacity in plant matrices. The results of the antioxidant capacity of the alcoholic extract’s study are shown in Table 1, and they vary between 58.60 ± 1.32 (*Mentha pulegium*)—81.65 ± 1.82 (*A. absinthium*) mmol Trolox/g sample and the percentage inhibition was 1.08% ± 0.03 (*Populus nigra*)—1.82% ± 0.05 (*Althaea officinalis*, *Origanum vulgare*).

The highest percentage of inhibition of the DPPH radical was registered for the extract of *Artemisia absinthium* extract, which have also a very good antibacterial activity.

### 2.3. Determination of Mineral Content

Analyzing the mineral potential of the plant samples a significant variation in Ca was found in *Evernia prunastri* (10,287.02 ± 17.32 μg/kg), Mg in *Melissa officinalis* (2357.07 ± 11.63 μg/kg), Fe in *Mentha pulegium* (1717.64 ± 17.52 μg/kg), Na in *Origanum vulgare* (1445.66 ± 8.31 μg/kg), and K in *Lavandula angustifolia* (23,443.88 ± 16.52 μg/kg) (Table 2).

On the other hand, according to Table 2. the content of microelements, and implicitly the contaminants from the studied plants show some inadequate values that were obtained with Cd: 90.44 ± 2.72 μg/kg (*Althaea officinalis*). In this case, the Cd concentration exceeds the maximum admissible limits [32,33]. Other slightly toxic elements (Pb, Ni, Cr) can be found in small quantities in all analyzed plants.

### 2.4. Antibacterial Activity

An antibacterial activity of the plant alcoholic extract was recorded for all of the bacterial strains and for all extracts with few exceptions. Six bacterial stains were resistant to different plant extracts: one of each *B. cereus* to *M. officinalis*; *E. coli* to *P. lanceolate*; *S. liquefaciens* to *A. absinthium*; *S. intermedius* and *Y. ruckeri* to *O. vulgare* (Table 3 and Table 4). However, more bacterial strains, especially the Gram negative ones, have shown antimicrobial resistance for conventional antibiotics such as penicillin (13/14), amoxicillin (10/14), and oxytetracycline (8/14) (Table 3 and Table 4). *Enterobacter intermedius* presented antimicrobial resistance also for florfenicol. Among Gram positive bacteria antimicrobial resistance was observed for penicillin (5/18), amoxicillin (4/18) and *Bacillus cereus* was resistant also for Ceftriaxone. 

Means comparison by ANOVA test for the inequality of means have shown no significant difference between the negative control (96° ethanol) and *A. foeniculum* (*p* = 0.07), penicillin (*p* = 0.66), and amoxicillin (*p* = 0.33). The higher inhibition area was observed for *E. prunastri*, closely followed by *A. absinthium*, without significant differences between them (*p* = 0.87). *Evernia prunastri* had a lower mean of inhibition area when compared with Enroxil and Floron (*p* = 0.0005), similar with Ceftriaxone (*p* = 0.13), gentamcin (*p* = 0.56), oxytetracycline (*p* = 0.76), amoxicillin (*p* = 0.06), and higher than penicillin (*p* = 0.04). The antimicrobial effect of *A. absinthium* and *L. angustifolia* was statistically similar (*p* = 0.09), being lower than that observed for enrofloxacin and florfenicol (*p* = 0.002), and similar with the rest of the tested antibiotics (*p* = 0.07–0.71). The remaining plant extract, except for *A. foeniculum*, had a similar antimicrobial effect with a p value varying from 0.05 to 0.77, being comparable with the effect of amoxicillin (*p* = 0.55–0.92), penicillin (*p* = 0.45–0.8), and lower than that recorded for gentamicin (*p* = 0.0007–0.035), Ceftriaxone (*p* = 0.00002–0.0006), Floron (*p* = 0.00001), and enrofloxacin (*p* = 0.00001). In addition, *P. nigra*, *A. officinalis*, *M. officinalis,* and *N. cataria* presented a similar antimicrobial effect as oxytetracycline (*p* = 0.05–0.08).

**Figure 1:**
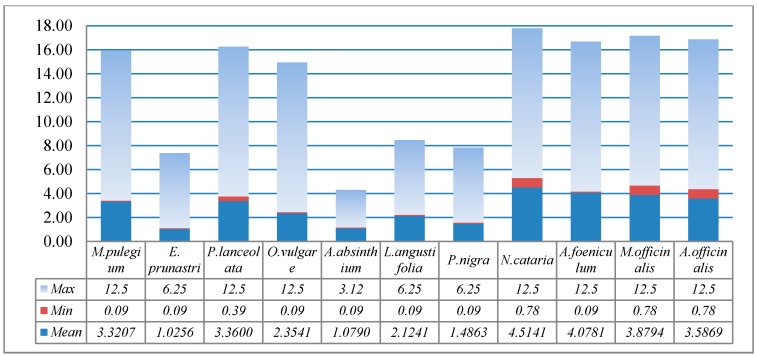
Minimum inhibitory concentrations (MIC) values (mg/mL) for 11 plant extracts.

**Figure 2:**
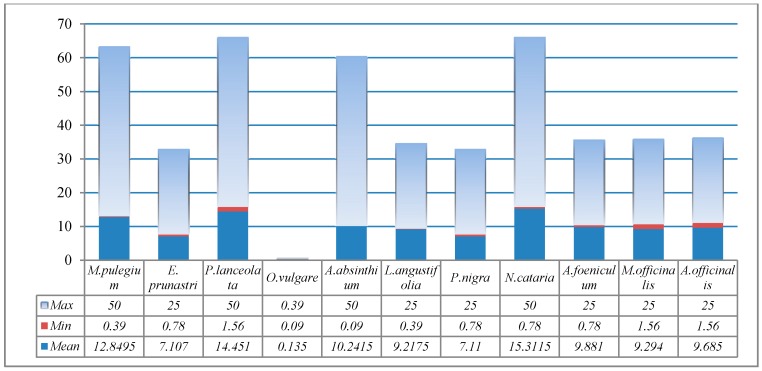
Minimum bactericidal concentration (MBC) values (mg/mL) for 11 plant extracts.

The MIC followed the same pattern with slight differences. *Evernia prunastri* had a MIC varying between 0.09 μL and 6.25 μL, with an average of 1.025, followed by *A. absinthium* (0.09–3.12 μL; 1.079), *P. nigra* (0.09–6.25 μL; 1.486), and *L. angustifolia* (0.09–6.25 μL; 2.124). The remaining plant extracts had recorded averages varying from 3.32 (*M. pulegium*) to 4.51 (*N. cataria*) (Figure 1). Similarly, small values of Minimum bactericidal concentration (MBC) and their averages were recorded for *E. prunastri* (0.75–25 μL; 7.11), *P. nigra* (0.75–25 μL; 7.11), and *L. angustifolia* (0.39–25 μL; 9.22). The remaining recorded MBC averages varied from 9.29 (1.56–25 μL) for *M. officinalis* to 15.31 (1.56–50 μL) for *N. cataria* (Figure 2).

The antimicrobial activity of biological products was assessed only by analysis of the inhibition of bacterial growth using the diffusion method. Among the tested bacterial strains, only two of them presented resistance, one *S. liquefaciens* at R5 and one *Vibrio fluvialis* at R4 and R6 (Table 3 and Table 4). The means of inhibition areas varied from 13.8 ± 5.9 (R5) to 17.1 ± 7.1 (R4), with few significant differences between the products (R1–R4 *p* = 0.02; R1–R7 *p* = 0.04, and R5–R7 *p* = 0.03). However, only R4, R3, and R7 presented means of inhibition areas comparable with both florfenicol and enrofloxacin, without significant differences between them (*p* value varied from 0.06 to 0.98). Also, for these products, a higher inhibition compared to penicillin (*p* = 0.00016, *p* = 0.00032, *p* = 0.00006), gentamicin (*p* = 0.00044, *p* = 0.00038, *p* = 0.00002), and amoxicillin (*p* = 0.00016, *p* = 0.00021, *p* = 0.00004) was observed.

## 3. Discussion

Historically, plant extracts or their derivate, have been used as a safe, effective, and natural remedy for aliments and diseases in traditional medicine. Moreover, in the past few decades, the search for new anti-infection agents has preoccupied many research groups in the field of ethno pharmacology. As a result, among 109 new antibacterial drugs, approved from 1981 to 2006, 69% originated from natural products [34]. However, the continued and further exploration of plant antimicrobials needs to be carried out in order to use more economic alternatives based on flora specific to a certain geographical area. While in North America, it is estimated that more than 2500 plant species are used as drugs [35], in Europe, only 590 plant species referring to 102 different plant families are reported to be used for animal treatment, with *Asteraceae*, *Fabaceae*, and *Lamiaceae* being the most important families [36].

The present study increases the number of data regarding the antimicrobial activity of plants originating from Southeastern Europe on pathogens isolated from BM. The obtained results have shown an inhibition of bacterial growth for all tested plants, with better results for *E. prunastri*, *A. absinthium*, and *L. angustifolia*. These results could be explained by the active compounds determined in these plants. Our result highlights a high amount of phenolic compounds and a high antioxidant activity of *A. absinthium*. The obtained values (Table S4) are consistent with the data found in literature. In this respect, the total polyphenols of the alcoholic extracts in plants as compared to the data obtained is 13.4 ± 0.2 mg·GAE/g DW in *Mentha pulegium*, 11.7 ± 1.1 mg·GAE/g DW to *Echinacea purpurea*, 2.7 ± 0.2 mg·GAE/g DW *Althaea officinalis*, and 8.0 ± 0.1 mg·GAE/g DW *Thymus vulgaris* [37,38]. Mihaylova et al. [39] compared the concentration of total polyphenols to various *Nepeta cataria* extracts, obtaining similar results with the present alcohol extract values (29.8 ± 0.3 mg·GAE/g DW).

Plants polyphenols are known to possess antibacterial activity. Stagos et al., [40] showed that the polyphenolic extracts from *Salvia*, *Mentha,* and *Sideritis* species have strong free radical scavenging activity and exhibit inhibitory activity against *S.aureus* growth.

Also, the content of microelements may influence the bacterial growth. Sordillo et al., [41] used plants with significant potential of Se, Cu, and Zn in the food of animals for the prevention of various diseases such as clinical and subclinical mastitis. Our results have shown a high concentration of Cu (259.36 ± 1.24 μg/kg) and Se (517.28 ± 11.29 μg/kg) for *Evernia prunastri*; and Zn (67.54 ± 0.17 μg/kg) for *Populus nigra*. *Lavandula angustifolia* instead, exhibited a high amount of K (23,443.88 ± 16.52 μg/kg). Potassium is an important mineral; its deficiency reduces mechanical stability, nutritional quality, and crop resistance to pathogens [42]. The leaves and stems of *Ocimum Gratissimum* (Laminaceae family) present as dietary intake are good sources of potassium, copper, iron, manganese, and zinc [43].

Throughout the ages, lichens have been used for various purposes, including folk medicines. Species of *Evernia*, *Peltigera*, *Parmelia*, *Cladonia*, *Rocella,* and *Pertusaria* were used to control fever, diarrhea, infections, skin diseases, epilepsy, convulsions, and as purgative [44]. The various beneficial effect of *E. prunastri* includes antioxidant, anticancer, antimicrobial [45], and antifungal [46] properties. *Evernia prunastri* have shown antimicrobial properties on several bacteria including *S. pyogenes*, *S. aureus*, *Bacillus* spp., *Enterococcus* spp., and *E. coli* [47,48]. However, for the majority of bacterial strains used in these studies there were not observed a resistance to the antibiotics used as positive controls. In our study, an important number of strains presented susceptibility to *E. prunastri*, but resistance to conventional antibiotics, which highlights the increasing multidrug resistance of pathogens isolated from BM, probably due to intramammary excessive antibiotic treatment.

*Artemisia absinthium,* best known as the principal ingredient in the infamous Absinthe drink, has been used medicinally since the times of ancient Greece, and also in western European systems of traditional medicine [49]. Aerial parts and leaves of *A. absinthium* (wormwood) are used in against ecto- and endoparasites, mainly in cattle. In vitro and in vivo data suggest that wormwood might be effective as an antihelmintic, but its use as an ectoparasitic or repellent agent needs to be substantiated [36]. It is also used in Romanian folk medicine as treatment for diarrhea in cattle and horse [50]. It was recently reported that the essential oils occurring in flowers and aerial parts from *A. absinthium* have also antimicrobial properties [49]. Several studies have shown an important anti-microbial growth inhibition of *A. absinthium* on diverse Gram positive and negative bacteria [51,52,53], including *S. aureus* isolated from BM [54].

Essential oils distilled from members of the genus *Lavandula* have been used both cosmetically and therapeutically for centuries. It is suggested that lavender oil is antibacterial, antifungal, carminative, sedative, antidepressive, and effective for burns and insect bites. Primarly, *L. angustifolia* has been found to have an inhibitory effect against many species of bacteria [23,55,56,57,58,59], including bacteria isolated from BM [60]. It has also been suggested that essential oils from lavender may be useful in treating bacterial infections that are resistant to antibiotics, such as methicillin-resistant *Staphylococcus aureus* and vancomycin-resistant *Enterococcus faecalis* [61].

Based on these plants and others of which a antimicrobial effect was observed in previous published research [27,33,60], eight recipes of plant derived products have been established. Minimum inhibitory and bactericidal concentrations for plant extract were detected in order to choose the plants feasible for obtaining final products with a possible intramammary application. The effect of these products on bacterial growth was tested in vitro on pathogens isolated from BM, having as a further aim, after more testing, a possible applicability in this disease.

## 4. Materials and Methods

### 4.1. Plant Material and Alcoholic Extracts

The plant material used in the present study was harvested from Research field of University of Agricultural Sciences and Veterinary Medicine, Cluj-Napoca, (UASVM), Romania in 2015. A voucher specimen of every plant is deposited in Herbarium of the Department of Botany, University of Agricultural Sciences and Veterinary Medicine Cluj, Napoca, Romania.

The alcoholic extract of squaw mint (*Mentha pulegium* L., Lamiaceae family), catnip (*Nepeta cataria* L., Lamiaceae), lemon balm (*Melissa officinalis* L., Lamiaceae), anise hyssop (*Agastache foeniculum* L. Lamiaceae), lavender (*Lavandula angustifolia* Mill., Lamiaceae), oregano (*Origanum vulgare* L., Lamiaceae), marshmallow (*Althaea officinalis* L., Malvaceae), narrowleaf plantain (*Plantago lanceolate* L., Plantaginaceae), absinthe wormwood (*Artemisia absinthium* L. Asteraceae), black poplar buds (*Populus nigra* L., Salicaceae), plum lichen (*Evernia prunastri* (L.) Ach. (1810), and Parmeliaceae) was screened for antimicrobial activity using an agar diffusion technique against 32 microorganisms isolated from cows previously diagnosed BM (clinical or sub-clinical) including one reference strain.

Different parts of plants were harvested in the maximum period of their bioactive principles amounts (Table S1), dried in the shade and in an airy place. The alcoholic extracts were obtained from 5 g of plant powder (grinded in fine powder) and 100 mL ethanol 96°. The flasks were kept for 14 days, protected from light and at the room temperature, during which the content was stirred daily. After 14 days, the extracts were filtered through filter paper and the content was brought to 100 mL with ethanol 96 °C, and the extract was placed in dark bottles at 4 °C until further uses. 

### 4.2. Bacterial Strains

The bacterial strains included in this study were morphologically identified based on cultural characteristics and bacteria morphology in Gram stained smears and by API test (Table S2). In total, seven *Staphylococcus xylosus*, four *S. intermedius*, one of each *S. chromogenes*, *S. hyicus*, *S. aureus*, seven *Vibrio fluvialis*, two *Serratia liquefaciens*, two *Escherichia coli*, two *Lactococcus lactis* ssp. *lactis*, and one of each *Enterobacter intermedius*, *Bacillus cereus*, *Yersinia ruckeri*, *Aeromonas hydrophila/caviae*, and *Kytococcus sedentarius* were included in this study. In total, 14 Gram negative and 18 Gram positive bacterial strains were included in the study (Table S2).

### 4.3. Chemicals

Phenolic acids and flavones (gallic and quercetin) were purchased from Karl-Roth, Karlsrue, Germany, Sigma-Aldrich, St. Louis, MO, USA, Fluka Switzerland. Other chemicals (2,2-diphenyl-1-picrylhydrazyl (DPPH), 6-hydroxy-2,5,7,8—tetramethylchroman-2-carboxylic acid (Trolox)) were purchased from Sigma-Aldrich, St. Louis, MO, USA. Matrix modifier for graphite furnace AAS (Pd(NO_3_)_2_/HNO_3_ ca. 15% (Palladium matrix modifier), Mg(NO_3_)_2_·6H_2_O in HNO_3_ ca. 17% (Magnesium matrix modifier), NH_4_H_2_PO_4_ 100 ± 2 g/L in H_2_O (Phosphate modifier) were purchased from Merck KgaA, 6471 Darmstadt, Germany. Standards solution (Cu(NO_3_)_2_) in HNO_3_ 0.5 mol/L (Copper standard solution), Cd(NO_3_)_2_ in HNO_3_ 0.5 mol/L (Cadmium standard solution), Cr(NO_3_)_3_ in HNO_3_ 0.5 mol/L (Chromium standard solution), Mn(NO_3_)_2_ in HNO_3_ 0.5 mol/L (Manganese standard solution), SeO_2_ in HNO_3_ 0.5 mol/L (Selenium standard solution), NaNO_3_ in H_2_O (Sodium standard solution), Mg(NO_3_)_2_ in HNO_3_ 0.5 mol/L (Magnesium standard solution), KNO_3_ in HNO_3_ 0.5 mol/L (Potassium standard solution), Fe(NO_3_)_3_ in HNO_3_ 0.5 mol/L (Iron standard solution), Ca(NO_3_)_2_ in HNO_3_ 0.5 mol/L (Calcium standard solution), Ni(NO_3_)_2_ in HNO_3_ 0.5 mol/L (Nickel standard solution), Pb(NO_3_)_2_ in HNO_3_ 0.5 mol/L (Lead standard solution), and Zn(NO_3_)_2_ in HNO_3_ 0.5 mol/L (Zinc standard solution) were purchased from Merck KgaA, Darmstadt, Germany.

### 4.4. Polyphenolic and Flavones Contents of the Extracts

For the total polyphenol content determination, the Folin-Ciocâlteu method was used [62], modified by various authors and adapted to all types of matrices [29,39,63,64,65]. A volume of 25 μL of each ethanolic extract was mixed for 5 min with 125 μL of 0.2N Folin-Ciocâlteu. The samples were incubated in the dark for 30 min. The absorbance was measured at 760 nm, using a Sinergy 2 Biotek Multichannel spectrophotometer. The standard curve was prepared by using different concentrations of gallic acid, and the absorbances were measured at 760 nm. TPC values were determined by using an equation obtained from the calibration curve of gallic acid graph (y = 11.63x + 0.0376, R^2^ = 0.9996). Total polyphenolic content was expressed as mg gallic acid/g dry material plant (mg GAE/g plant material).

The quantification of the flavones in the samples was made using Dowd method [66], based on the reaction of a solution of aluminium chloride, as specific reagent, with the flavonoids present in the sample giving a yellow color, with the intensity of this color being determined spectrophotometrically at 415 nm.

Each extract (150 μL) was mixed with aluminium chloride (150 μL, 2%), diluted in methanol. The calibration curve, different concentrations of quercetin (0.005–0.12 mg/mL), subjected to the same steps as the extracts, was used for flavonoid determination (y = 37.125*x* − 0.0328, R^2^ = 0.9997). Results were expressed as quercetin equivalents. 

### 4.5. Radical Scavenging Activity Assay (DPPH)

The antioxidant activity is the first step in assessing the biological activity of any matrix. DPPH free radical method is an antioxidant assay based on electron- transfer that produces a violet solution in ethanol. This free radical, stable at room temperature, is reduced in the presence of an antioxidant molecule, giving a discoloration of solution, proportionally with the amount of antioxidant present in the sample [29,63]. The free radical scavenging ability of the ethanolic extracts was measured in terms of hydrogen donation or radical scavenging ability using this method. Thus, 5 μL of 1% plant alcoholic extract was mixed with 295 μL of 0.02 mg/mL DPPH solution in methanol, stirred and incubated in the dark for 20 min. The absorbance changes were monitored at 517 nm using a Sinergy 2 Biotek multichanel spectrophotometer. The percentage of DPPH consumption in each case was converted to Trolox equivalents using a calibration curve (y = 680.08x + 1.525, R^2^ = 0.9959) with different Trolox concentrations (0.008–0.1 mmol/L). The percent inhibition of DPPH free radical was calculated by the formula:

Percentage inhibition (%)= [(A_blank_ − A_sample_)/A_blank_] × 100

where, A_blank_ is the absorbance of the control reaction (DPPH alone), and A_sample_ is the absorbance of DPPH solution in the presence of the test compound.

### 4.6. Determination of Mineral Content by Atomic Absorption Spectroscopy

To determine the levels of micro and macroelements: Ni, Na, Cd, Mg, K, Cr, Ca, Fe, Mn, Cu, Se, Pb, and Zn from studied plant matrices, the atomic absorption spectrometry method was used.

The mineralization of the samples was performed in a microwave furnace, Berghof digestion system MWS-2. Approximately 0.3 g of the homogenized plant samples were placed in special Teflon tubes, 2 mL of 65% HNO_3_ was added and let to react for 15 min, after which 3 mL of H_2_O_2_ was added before the container was sealed [33,67,68,69].

At the end of the initiated program, the solution is transferred into graded plastic containers and the sample is diluted with ultrapure water to a volume of 125 mL.

An Aanalyst 800 Atomic Absorption Spectrometer from Perkin-Elmer (Shelton, CT, USA) was used, equipped with a cross-linked graphite furnace. It is electrically heated, and the voltage will be applied transversely to the tube, perpendicular to the light beam, and finally the electromagnet will generate a magnetic field parallel to the radiation beam emitted by the lamp [68].

The reagents used to perform the analyses were of analytical purity, namely 65% nitric acid and ultra-purified water. For the calibration curve, following dilutions of standard solutions were used: chromium (10 μg/L), manganese (10 μg/L), calcium (2 μg/L), nickel (50 μg/L), iron (20 μg/L), cadmium (2.0 μg/L), sodium (4.0 μg/L), potassium (5.0 μg/L), zinc (2.0 μg/L), selenium (100 μg/L), magnesium (1.0 μg/L), copper (25 μg/L), and lead (50 μg/L) [33,68].

The result obtained represents the concentration of mineral elements expressed in μg/L, than transformed according to the amount of weighted sample.

### 4.7. Antibacterial Activity Determination 

For the antibacterial effect of vegetal extracts, the model of bacteria sensibility to antibiotics and chemotherapeutic using the diffusion method in Mueller Hinton agar was used [70]. For this purpose, Petri dishes of 9 cm diameter were used and about 15 mL of sterile medium. From each tested strain, a suspension was prepared by inoculating a quantity of bacterial strain (from one colony) grown on an agar plate with sheep blood, in 10 mL of saline until the bacterial density coincides to 0.5 McFarland scale (1–3 × 108 CFU (Colonies forming units)/mL). Petri plates were floated with the suspension and then dried. Microcomprimats were made from special filter paper in the lab, using an eyelet of 5 mm, were sterilized in UV light for 30 min and then loaded with 50 µL plant extract or plant based product. Nine micro-comprimats (discs of filter paper) with plant extracts and products were distributed around each plate. Incubation was performed at 37 °C for 24 h, and the results were expressed in mm zone of inhibition. The means, Standard Deviation (SD), and the significance of main differences were calculated in EpiInfo 7 (CDC) software. *p* values of < 0.05 were considered as significant.

Minimum inhibitory concentrations (MIC) were determined by the serial dilution method. For this analysis, 100 μL nutrient broth was placed in a 96 well plate and 100 μL of the analyzed plant extract were added in the first ten lines. Then 100 μL were aspirated from every well and placed in the second well line of the plate. This technique was used to obtain the desired dilutions until the line 10 and from the last well, 100 μL mixes was discharged resulting in the following concentration: 50 μL, 25 μL, 12.5 μL, 6.25 μL, 3.12 μL, 1.56 μL, 0.78 μL, 0.39 μL, 0.19 μL, and 0.09 μL of active substance in 100 μL medium. Each well was seeded with 5 μL of a 24 h culture bacterial suspension, adjusted to be similar to 0.5 McFarland scale, and incubated for 24 h at 37 °C. MIC was detected by the lowest concentration of the analyzed product, in which the development of the bacterium strain was inhibited (medium remained clear). Negative control (ethanol) concentration for the determination of MIC using serial dilution method, was similar with the concentration used in the 1st well of the plate.

Minimum bactericidal concentration (MBC) was determined by cultivating each suspension in which was observed an inhibition at the MIC test on nutrient broth agar for 2 4h at 37 °C. MBC was detected by the lowest concentration of the analyzed suspension, of which no colonies were obtained.

### 4.8. Plant Biological Products

Based on the results obtained in this study and from previous studies performed in the same laboratory [27,33,63], eight biological products were produced using different concentrations of mentioned plant extracts (Table S3).

### 4.9. Statistical Analysis

Statistical differences between the means were estimated using ANOVA (parametric test for inequality of means). Probability value of *p < 0.05* was considered to be statistically significant. The means, Standard Deviation (SD), and the significance of main differences were calculated in Epi Info^TM^ 7 (CDC, Atlanta, GA, USA) software. Quantitative data matrix was processed in Microsoft Excel 2010 (Microsoft) for statistical calculations (mean and standard deviation).

## 5. Conclusions

The results obtained for three products have shown a strong anti-microbial effect comparable with the effect of florfenicol and enrofloxacin, and better than individual plant extracts possibly due to synergism. To the best of our knowledge, this is one of the few studies regarding the anti-microbial effect of plant-derived products on pathogens isolated in BM conducted in Europe. However, further in vivo testing and acute toxicity studies are required for the safe use of these products.

## Figures and Tables

**Table 1:** Total Polyphenolic and Flavones Content, Radical scavenging activity of studied the plant extracts. Results are given as mean ± SD.

Plant Extracts	Polyphenols (mg·GAE/g DW)	Flavones (mg·QE/g DW)	Inhibition Percentage (%)	RSA (Mmoli Trolox/g)
Mp	30.2 ± 0.84	150.6 ± 1.51	58.60 ± 1.32	1.24 ± 0.03
Pn	2.4 ± 0.45	20.8 ± 0.95	70.01 ± 1.13	1.08 ± 0.03
Ao	3.3 ± 0.61	16.5 ± 0.67	74.95 ± 0.90	1.82 ± 0.03
Pl	6.1 ± 0.76	31.0 ± 0.55	77.66 ± 1.43	1.56 ± 0.04
Ov	14.3 ± 0.77	32.1 ± 0.56	79.88 ± 1.95	1.82 ± 0.05
Ep	14.2 ± 0.56	74.1 ± 1.21	70.40 ± 0.98	1.65 ± 0.02
La	15.7 ± 0.91	34.6 ± 0.44	77.76 ± 1.02	1.51 ± 0.04
Nc	18.1 ± 0.68	4.8 ± 0.22	77.92 ± 1.95	1.52 ± 0.03
Mo	26.5 ± 0.87	14.8 ± 0.34	74.19 ± 1.85	1.72 ± 0.02
Aa	79.2 ± 1.36	9.1 ± 0.24	81.65 ± 1.82	1.69 ± 0.03
Af	45.5 ± 1.02	89.0 ± 1.01	80.79 ± 1.91	1.75 ± 0.03

Mp—*Mentha pulegium*; Nc—*Nepeta cataria*; Mo—*Melissa officinalis*; Af—*Agastache foeniculum*; La—*Lavandula angustifolia*; Ov—*Origanum vulgare*; Ao—*Althaea officinalis*; Pl—*Plantago lanceolata*; Aa—*Artemisia absinthium*; Pn—*Populus nigra*; Ep—*Evernia prunastri*; RSA—Radical Scavenging Activity.

**Table 2:** Macroelements and microelements content of plant samples. Results are given as mean ± SD.

*Sample*	Ca (µg/kg)	Mg (µg/kg)	Fe (µg/kg)	Na (µg/kg)	K (µg/kg)	Cr (µg/kg)	Cd (µg/kg)	Ni (µg/kg)	Mn (µg/kg)	Cu (µg/kg)	Se (µg/kg)	Pb (µg/kg)	Zn (µg/kg)
Mp	4630.15 ± 17.59	504.82 ± 1.29	1717.64 ± 17.52	0	632.73 ± 3.95	88.64 ± 4.26	42.18 ± 5.31	35.75 ± 0.61	84.83 ± 1.56	121.14 ± 1.35	181.02 ± 4.57	3.68 ± 0.23	51.17 ± 0.17
Pn	6749.50 ± 39.27	539.84 ± 1.41	1478.07 ± 14.10	12.38 ± 0.24	641.17 ± 6.88	77.65 ± 1.83	41.29 ± 7.31	19.26 ± 0.32	245.18 ± 4.37	118.52 ± 7.52	185.59 ± 8.22	2.18 ± 0.27	67.54 ± 0.17
Ao	6963.69 ± 17.65	535.86 ± 2.72	303.99 ± 4.78	0	644.24 ± 9.42	117.67 ± 3.30	90.44 ± 2.72	11.04 ± 0.41	262.63 ± 4.85	177.42 ± 9.48	107.88 ± 14.16	0.68 ± 0.2	64.76 ± 0.53
Pl	7098.04 ± 12.95	535.42 ± 2.05	56.21 ± 8.28	165.45 ± 11.18	630.78 ± 9.00	32.79 ± 1.14	36.06 ± 9.61	12.86 ± 0.01	199.29 ± 1.63	46.81 ± 5.24	190.16 ± 0.35	5.88 ± 0.78	29.63 ± 0.22
Ov	5071.45 ± 10.39	501.47 ± 2.65	0	1445.66 ± 8.31	552.18 ± 1.17	37.94 ± 1.29	45.66 ± 4.31	9.48 ± 0.49	244.90 ± 4.82	146.85 ± 8.64	40.10 ± 9.63	4.50 ± 0.49	25.87 ± 0.35
Ep	10,287.02 ± 17.32	418.76 ± 1.71	1653.08 ± 11.44	103.99 ± 5.88	378.45 ± 12.59	123.85 ± 5.66	13.99 ± 3.88	15.38 ± 0.2	237.38 ± 2.64	259.36 ± 1.24	517.28 ± 11.29	0	36.22 ± 0.13
La	2027.21 ± 12.35	1420.07 ± 10.71	323.13 ± 7.89	0	23,443.88 ± 16.52	0	0	11.05 ± 0.98	14.12 ± 1.76	13.44 ± 1.26	85.6 ± 9. 02	0	20.83 ± 1.11
Nc	4836.56 ± 11.13	2113.91 ± 11.23	73.63 ± 3.45	0	20,013.34 ± 15.78	0	0	9.09 ± 0.55	20.76 ± 1.85	10.09 ± 0.35	35.55 ± 1.01	0	26.13 ± 0.96
Mo	4064.49 ± 8.32	2357.07 ± 11.63	69.10 ± 5.67	0	18,911.22 ± 16.35	0	0	8.96 ± 0.63	33.42 ± 1.79	10.30 ± 0.29	21.22 ± 0.87	0	24.10 ± 1.34
Aa	9005.82 ± 5.88	867.72 ± 11.77	97.25 ± 9.15	0	20,991.05 ± 14.47	0	0	11.70 ± 1.21	46.54 ± 1.39	120.78 ± 2.56	212.34 ± 4.06	0	35.63 ± 0.86
Af	9505.12 ± 19.58	491.85 ± 3.53	3.87 ± 0.65	0	19,984.98 ± 14.82	0	0	1.10 ± 1.12	63.75 ± 1.75	79.44 ± 1.59	246.62 ± 1.65	0	26.78 ± 0.35

Mp—*Mentha pulegium*; Nc—*Nepeta cataria*; Mo—*Melissa officinalis*; Af—*Agastache foeniculum*; La—*Lavandula angustifolia*; Ov—*Origanum vulgare*; Ao—*Althaea officinalis*; Pl—*Plantago lanceolata*; Aa—*Artemisia absinthium*; Pn—*Populus nigra*; Ep—*Evernia prunastri*.

**Table 3:** Antimicrobial activity of plant extracts, negative control and antibiotics used, (mm, zone of inhibition).

Species	Mp	Nc	Mo	Af	La	Ov	Ao	Pl	Aa	Pn	Ep	C	O	C	G	P	F	E	A
*A. hydrophila caviae*	6.0	11.0	11.0	6.0	8.0	8.5	7.0	8.0	13.0	7.0	14.0	0.0	0.0	20.9	8.5	0.0	19.7	19	0.0
*B. cereus*	8.0	8.0	0.0	7.5	10.0	12.0	10.0	6.0	20.0	8.0	13.0	8.0	12.7	0.0	7.6	0.0	17.1	13.1	0.0
*E. intermedius*	8.0	10.5	10.0	9.0	13.0	8.0	9.0	11.0	7.0	9.5	13.0	8.5	10.7	6.7	13.3	0.0	0.0	20.2	11.3
*E. coli*	7.0	10	9.0	9.0	12.0	8.0	6.0	0.0	7.0	7.0	22.0	0.0	14.4	0.0	7.5	0.0	12.3	18.3	7.7
*E. coli*	10.0	8.5	12.0	9.0	12.5	7.0	12.0	13.0	6.0	9.0	9.0	8.0	20.6	8.5	14.5	0.0	22.7	19.6	0.0
*K. sedentarius*	8.0	10.0	10.0	7.0	7.5	7.5	7.0	8.0	14.5	12.0	19.0	6.0	22.2	16.1	15.5	21.8	17.1	19.2	13.9
*L. lactis*	8.0	7.5	7.0	10.0	7.5	8.0	9.0	7.0	10.0	6.0	10.0	8.0	19.6	12.2	14.1	16.2	14.7	13.8	19.9
*L. lactis*	8.0	10.0	8.0	8.5	10.0	11.0	8.0	8.5	12.0	12.0	21.0	8.0	21.6	10.9	14.7	21.7	20.6	18.2	18.2
*S. liquefaciens*	10.0	11.0	11.0	9.0	10.0	10.0	16.0	13.0	11.0	10.0	10.0	6.0	0.0	18.9	9.5	0.0	17.8	11.0	0.0
*S. liquefaciens*	7.0	9.0	9.0	7.0	8.0	8.0	7.0	7.0	0.0	11.0	10.0	8.0	0.0	17.4	9.9	0.0	18.3	18.8	0.0
*S. aureus*	6.0	8.0	11.0	8.5	10.0	9.0	7.0	9.0	23.0	9.0	8.0	8.5	17.8	13.3	9.5	25.1	15.3	17.4	23.5
*S. chromogenes*	7.5	8.0	9.0	8.0	10.0	11.0	8.5	8.5	12.0	9.5	12.0	8.0	21.4	11.6	16.6	20.9	18.1	22.2	14.4
*S. hyicus*	8.5	10.0	11.0	9.0	10.0	12.0	8.5	11.5	9.5	11.5	9.0	9.0	17.5	11.7	12.6	21.7	21.9	21.5	20.6
*S. intermedius*	11.5	11.0	9.5	8.5	9.0	8.5	10.0	8.0	10.0	12.0	9.5	8.0	17.1	24.1	8.7	0.0	19.2	18.7	0.0
*S. intermedius*	8.5	8.5	9.5	10.0	10.0	0.0	11.0	10.0	10.5	10.0	20.0	8.0	16.7	9.5	12.4	21.5	19.5	20.8	19.5
*S. intermedius*	8.5	10.0	11.0	11.5	10.0	11.5	7.0	8.5	9.0	9.5	11.5	8.5	15.9	14.1	5.8	0.0	17.4	14.7	0.0
*S. intermedius*	9.0	11.0	10.0	10.0	11.0	9.0	8.0	11.5	8.0	9.5	12.5	8.0	17.2	10.4	12.4	22.8	18.1	21.2	19.6
*S. xylosus*	8.0	12.5	12.5	7.0	11.5	7.5	16.0	12.0	20.0	14.0	8.0	8.5	23.7	14.9	15.9	6.2	18.9	18.1	10.3
*S. xylosus*	9.0	9.0	11.0	6.0	9.0	18.5	10.0	7.5	14.0	13.0	12.0	8.5	23.8	14.1	15.4	22.1	15.9	19.9	19.2
*S. xylosus*	10.0	8.5	11.5	9.0	10.0	8.0	10.0	8.0	7.0	9.0	12.0	8.5	15.1	18.5	10.1	0.0	21.5	21.4	0.0
*S. xylosus*	13.0	11.5	12.0	10.0	11.0	11.0	10.0	10.0	8.5	13.5	12.0	10.0	19.3	17.4	6.8	0.0	18.8	17.4	10.9
*S. xylosus*	13.0	8.5	9.0	9.5	10.5	8.0	13.5	9.0	12.0	9.0	10.0	10.0	23.2	11.1	11.4	7.08	0.0	13.1	11.1
*S. xylosus*	0.0	10.0	8.0	7.0	11.0	10.0	8.0	6.0	20.0	8.0	12.0	7.0	13.3	6.7	18.4	13.2	15.3	20.5	19.4
*S. xylosus*	7.0	11.0	10.0	8.0	12.0	6.0	9.0	9.0	14.5	9.0	12.0	8.0	13.6	5.76	17.61	9.6	26.1	18.9	16.5
*V. fluvialis*	6.0	8.0	10.0	6.0	11.0	12.0	12.0	10.0	8.0	9.0	14.0	8.5	0.0	17.6	9.2	0.0	18.6	18.9	0.0
*V. fluvialis*	10.0	8.0	7.0	9.0	9.0	10.5	11.0	9.0	10.0	10.0	11.0	7.0	9.9	27.7	14.8	0.0	25.4	25.7	0.0
*V. fluvialis*	11.0	10.0	12.0	8.0	12.0	12.0	13.0	8.0	17.0	9.0	12.0	8.0	0.0	20.1	10.9	0.0	10.3	19.2	0.0
*V. fluvialis*	10.0	8.0	7.5	7.0	10.0	12.0	8.0	7.0	20.5	13.0	11.0	7.0	0.0	20.9	6.2	0.0	9.9	10.8	0.0
*V. fluvialis*	7.0	9.5	10.0	7.0	11.0	8.0	12.0	11.0	19.5	9.0	8.0	7.0	0.0	20.1	7.2	0.0	6.2	16.2	0.0
*V. fluvialis*	11.0	10.0	8.5	7.0	9.0	8.0	10.0	9.0	0.0	7.0	9.0	0.0	0.0	19.2	9.2	0.0	18.2	16.3	9.1
*V. fluvialis*	12.5	10.5	10.0	8.0	10.0	10.5	10.0	13.0	24.0	11.0	12.5	9.5	26.7	20.1	22.3	30.3	32.1	28.1	23.1
*Y. ruckeri*	8.5	10	8.5	8.0	10.0	0.0	8.0	7.5	7.0	11.0	12.0	7.5	16.5	14.2	6.3	0.0	19.5	19.7	0.0
**Mean ± SD**	8.6 ± 2.5	9.6 ± 1.3	9.5 ± 2.3	8.3 ± 1.34	10.2 ± 1.4	9.1 ± 3.4	9.7 ± 2.5	8. 9 ± 2.5	12.0 ± 6.3	9.9 ± 1.9	12.2 ± 3.6	7.3 ± 2.5	12.7 ± 8.9	14.2 ± 6.4	11.7 ± 4.1	8.1 ± 10.4	17.1 ± 6.6	18.5 ± 3.7	8.9 ± 8.8

Mp—*Mentha pulegium*; Nc—*Nepeta cataria*; Mo—*Melissa officinalis*; Af—*Agastache foeniculum*; La—*Lavandula angustifolia*; Ov—*Origanum vulgare*; Ao—*Althaea officinalis*; Pl—*Plantago lanceolata*; Aa—*Artemisia absinthium*; Pn—*Populus nigra*; Ep—*Evernia prunastri*; C—negative control ethanol 96°; O—oxytetracycline; C—ceftriaxone; G—gentamicin; P—penicillin; F—florfenicol; E—enrofloxacin; and, A—amoxicillin.

**Table 4:** Antimicrobial activity of developed products and antibiotics used, (mm, zone of inhibition).

Species	R1	R2	R3	R4	R5	R6	R7	R8	O	C	G	P	F	E	A
*A. hydrophila caviae*	7.3	6.9	7.5	6.1	5.7	8.1	9.7	8.3	0	20.9	8.5	0.0	19.7	19.0	0.0
*B. cereus*	20.5	19.1	20.6	21.7	11.2	19.9	22.5	17.5	12.7	0.0	7.6	0.0	17.1	13.1	0.0
*E. intermedius*	8.6	7.1	10.8	13.1	7.6	10.2	11.3	12.7	10.7	6.7	13.3	0.0	0	20.2	11.3
*E. coli*	10.1	5.9	9.3	12.6	13.7	7.5	13.2	9.9	14.4	0.0	7.5	0.0	12.3	18.3	7.7
*E. coli*	11.3	6.3	9.1	11.9	12.9	7.9	12.9	10.2	20.6	8.5	14.5	0.0	22.7	19.6	0.0
*K. sedentarius*	20.1	21.5	25.1	25.3	20.2	21.9	27.3	20.3	22.2	16.1	15.5	21.8	17.1	19.2	13.9
*L. lactis*	16.9	22.2	22.3	25.7	18.8	16.44	18.3	19.2	19.6	12.2	14.1	16.2	14.7	13.8	19.9
*L. lactis*	17.1	21.8	21.5	23.9	21.2	18.2	23.7	22.1	21.6	10.9	14.7	21.7	20.6	18.2	18.2
*S. liquefaciens*	5.8	8.5	12.3	10.8	0.0	12.1	9.1	10.1	0.0	18.9	9.5	0.0	17.8	11	0.0
*S. liquefaciens*	8.9	7.1	10.9	13.4	7.9	10.4	11.2	12.1	0.0	17.4	9.9	0.0	18.3	18.8	0.0
*S. aureus*	17.1	17.5	13.9	17.8	14.9	13.9	14.2	16.1	17.8	13.3	9.5	25.1	15.3	17.4	23.5
*S. chromogenes*	13.9	17.1	21.5	17.2	17.1	15.4	18.6	12.3	21.4	11.6	16.6	20.9	18.1	22.2	14.4
*S. hyicus*	14.6	15.9	16.3	24.3	17.1	18.6	19.3	18.7	17.5	11.7	12.6	21.7	21.9	21.5	20.6
*S. intermedius*	7.3	10.9	15.2	10.4	6.7	14.1	14.4	12.3	17.1	24.1	8.7	0.0	19.2	18.7	0.0
*S. intermedius*	21.2	21.7	24.5	22.9	20.9	22.3	21.8	22.9	16.7	9.5	12.4	21.5	19.5	20.8	19.5
*S. intermedius*	18.5	19.6	20.4	22.2	16.67	13.3	20.6	18.9	15.9	14.1	5.8	0.0	17.4	14.7	0.0
*S. intermedius*	19.7	20.3	22.3	22.5	18.7	17.1	21.5	19.9	17.2	10.4	12.4	22.8	18.1	21.2	19.6
*S. xylosus*	16.7	18.1	18.1	23.4	23.2	14.2	14.2	14.1	23.7	14.9	15.9	6.2	18.9	18.1	10.3
*S. xylosus*	16.9	18.2	17.9	23.5	23.4	14.5	14.6	14.3	23.8	14.1	15.4	22.1	15.9	19.9	19.2
*S. xylosus*	16.5	17.9	18.2	23.3	23.1	14.1	14.2	14.1	15.1	18.5	10.1	0.0	21.5	21.4	0.0
*S. xylosus*	17.9	16.4	19.7	21.7	13.9	22.1	21.3	20.7	19.3	17.4	6.8	0.0	18.8	17.4	10.9
*S. xylosus*	18.2	20.8	21.5	24.1	17.7	17.9	21.8	20.4	23.2	11.1	11.4	7.08	0.0	13.1	11.1
*S. xylosus*	18.1	16.5	19.8	21.9	13.9	22.2	21.5	20.9	13.3	6.7	18.4	13.2	15.3	20.5	19.4
*S. xylosus*	18.1	20.1	21.2	24.1	17.7	17.8	21.6	20.7	13.6	5.76	17.61	9.6	26.1	18.9	16.5
*V. fluvialis*	5.51	5.8	6.3	5.9	6.3	9.4	11.1	11.9	0.0	17.6	9.2	0.0	18.6	18.9	0.0
*V. fluvialis*	5.6	7.8	8.1	5.5	7.8	8.1	11.2	10.7	9.9	27.7	14.8	0.0	25.4	25.7	0.0
*V. fluvialis*	5.5	9.2	9.4	0.0	9.2	0.0	11.3	9.6	0.0	20.1	10.9	0.0	10.3	19.2	0.0
*V. fluvialis*	15.4	12.6	14.3	18.1	12.7	12.8	17.9	12.8	0.0	20.9	6.2	0.0	9.9	10.8	0.0
*V. fluvialis*	13.2	15.1	17.9	10.8	9.3	18.3	19.3	13.5	0.0	20.1	7.2	0.0	6.2	16.2	0.0
*V. fluvialis*	10.6	5.9	9.1	12.3	13.9	7.8	13.4	9.8	0.0	19.2	9.2	0.0	18.2	16.3	9.1
*V. fluvialis*	20.5	19.4	20.9	21.5	11.7	19.8	22.9	17.5	26.7	20.1	22.3	30.3	32.1	28.1	23.1
*Y. ruckeri*	7.8	10.6	15.2	9.9	8.0	14.1	15.1	10.9	16.5	14.2	6.3	0.0	19.5	19.7	0.0
**Mean ± SD**	13.9 ± 5.2	14.5 ± 5.8	16.3 ± 5.5	17.1 ± 7.1	13.8 ± 5.9	14.4 ± 5.3	16.9 ± 4.9	15.2 ± 4.4	12.7 ± 8.9	14.2 ± 6.4	11.7 ± 4.1	8.1 ± 10.4	17.1 ± 6.6	18.5 ± 3.7	8.9 ± 8.8

R1–R8-Recipes for natural products (Table S1); O—oxytetracycline; C—ceftriaxone; G—gentamicin; P—penicillin; F—florfenicol; E—enrofloxacin; and, A—amoxicillin.

## Supplementary Materials

Supplementary materials are available online.
