# Supplementary material for: Medicinal Plants Based Products Tested on Pathogens Isolated from Mastitis Milk
Source: Molecules. 2017 Sep 4;22(9):1473. doi: 10.3390/molecules22091473 (PMC6151574; doi:10.3390/molecules22091473)
Supplement: Supplementary file 1 [file molecules-22-01473-s001.pdf]

Supporting Information

Table S1. Plant included in this study

Common name	Latin name	Part of the plant used	Harvesting month
Squaw mint	*Mentha pulegium*	leaves	July
Catnip	*Nepeta cataria*	stem, leaves, inflorescences	May
Lemon balm	*Melissa officinalis*	stem, leaves, inflorescences	August
Anise hyssop	*Agastache foeniculum*	stem, leaves, inflorescences	August
Lavender	*Lavandula angustifolia*	inflorescences	August
Oregano	*Origanum vulgare*	inflorescences	September
Marsh mallow	*Althaea officinalis*	inflorescences	July
Narrowleaf plantain	*Plantago lanceolate*	leaves	May
Absinthe wormwood	*Artemisia absinthium*	inflorescences	August
Black poplar buds	*Populus nigra*	buds	April
Plum lichen	*Evernia prunastri*	lichen	May

Table S2. The bacterial strains included in the study

**Species**	**Gram**	**Identification (API test and code)**	**Origin**
Aeromonas hydrophila/caviae	G-	API 20 NE V7.0, 5 4 7 7 7 4 4	Milk
Bacillus cereus	G+	API 50 CHB , 7212	Milk
Enterobacter intermedius	G-	API 20 E V4.1, 1 1 0 4 5 7 2	Milk
Escherichia coli 1,	G-	API 20 E, 7 1 0 4 5 7 2	Milk
Escherichia coli 1,	G-	API 20 E, 7 1 0 4 5 7 2	Milk
Kytococcus sedentarius	G+	API Staph, 0 0 1 0 0 0 0	Milk
Lactococcus lactis	G+	API 20 STREP, 7 0 0 5 5 1 1	Milk
Lactococcus lactis	G+	API 20 STREP, 7 0 0 5 5 1 1	Milk
Serratia liquefaciens	G-	API 20 E V4.1, 7 1 0 6 1 6 2	Milk
Serratia liquefaciens	G-	API 20 E V4.1, 6 1 0 6 1 6 1	Milk
Staphilococcus aureus	G+	ATCC 6538P	Reference
Staphylococcus chromogenes	G+	API Staph, 6712052	Milk
Staphylococcus hyicus	G+	API Staph V4.1, 6 7 3 2 1 5 3	Milk
Staphylococcus intermedius	G+	API Staph V4.1, 6 7 3 0 1 5 1	Milk
Staphylococcus intermedius	G+	API Staph V4.1, 6 7 3 0 1 5 1	Milk
Staphylococcus intermedius	G+	API Staph V4.1, 6 7 3 0 1 5 1	Milk
Staphylococcus intermedius	G+	API Staph V4.1, 6 7 3 0 1 5 1	Milk
Staphylococcus xylosus	G+	API Staph, 6 7 3 2 4 5 2	Milk
Staphylococcus xylosus	G+	API Staph, 6 7 3 2 4 5 2	Milk
Staphylococcus xylosus	G+	API Staph, 6 7 3 2 4 5 2	Milk
Staphylococcus xylosus	G+	API Staph V4.1, 6 7 7 3 1 5 1	Milk
Staphylococcus xylosus	G+	API Staph V4.1, 6 7 7 3 1 5 1	Milk
Staphylococcus xylosus	G+	API Staph V4.1, 6 7 3 2 5 5 3	Milk
Staphylococcus xylosus	G+	API Staph V4.1, 6 7 3 2 5 5 3	Milk
Vibrio fluvialis	G-	API 20 E V4.1, 6 1 0 6 1 2 0	Milk
Vibrio fluvialis	G-	API 20 E V4.1, 6 1 0 6 1 2 0	Milk

Vibrio fluvialis	G-	API NE, 7 1 1 6 1 2 2	Milk
Vibrio fluvialis	G-	API NE, 7 1 1 6 1 2 2	Milk
Vibrio fluvialis	G-	API NE, 7 1 1 6 1 2 2	Milk
Vibrio fluvialis	G-	API NE, 7 1 1 6 1 2 2	Milk
Vibrio fluvialis	G-	API 20 NE V7.0, 7 5 7 2 7 4 4	Milk
Yersinia ruckeri	G-	API 20 E V4.1; 6 1 0 4 1 2 0	Milk

Table S3. The recipe of biological products (100g)

Recipe	Active ingredients (g)	Excipients
R1	Propolis aq. 3, hops al. 2, plum lichen al. 7, lavender al. 4, black poplar buds al. 3, sage al. 3, marigold al. 4, oregano eo. 0.09, lavender eo. 0.09, rosemary eo. 0.09.	Xanthan gum (E 415) and water in a ratio of 1 to 200, Cosgard 0.6g
R2	Propolis aq. 10, *Magnum* hops al. 3, plum lichen al. 5, lavender al. 2, black poplar buds al. 4, sage al. 3, marigold al. 2, oregano eo. 0.09, lavender eo. 0.09, rosemary eo. 0.09.	
R3	Propolis aq. 3, *Brewers Gold* hops al. 3, hops pearls al. 3, plum lichen al. 5, common mallow al. 2, marigold al. 2, absinthe wormwood al. 5, black poplar buds al. 3, lemon balm al. 3, oregano eo. 0.09, lavender eo. 0.09, rosemary eo. 0.09.	
R4	Propolis aq. 5, *Brewers Gold* hops al. 2, hops pearls al. 2, plum lichen al. 3, common mallow al. 2, marigold al. 3, absinthe wormwood al. 7, black poplar buds al. 5, lemon balm al. 2, oregano eo. 0.09, lavender eo. 0.09, rosemary eo. 0.09.	
R5	Propolis aq. 7, hops al. 5, plum lichen al. 7, lavender al. 5, black poplar buds al. 7, sage al. 5, marigold al. 3, oregano eo. 0.09, lavender eo. 0.09, rosemary eo. 0.09.	
R6	Propolis aq. 10, hops al. 5, plum lichen al. 4, lavender al. 6, black poplar buds al. 5, sage al. 6, marigold al. 3, oregano eo. 0.09, lavender eo. 0.09, rosemary eo. 0.09.	
R7	Propolis aq. 7, *Brewers Gold* hops al. 3, hops pearls al. 3, plum lichen al. 5, common mallow al. 2, marigold al. 2, absinthe wormwood al. 8, black poplar buds al. 6, lemon balm al. 3, oregano eo. 0.09, lavender eo. 0.09, rosemary eo. 0.09.	
R8	Propolis aq. 5, *Brewers Gold* hops al. 3, hops pearls al. 3, plum lichen al. 7, common mallow al. 2, marigold al. 2, absinthe wormwood al. 8, black poplar buds al. 5, lemon balm al. 4, oregano eo. 0.09, lavender eo. 0.09, rosemary eo. 0.09.	

Aq.- aqueous extract; al.- alcoholic extract; eo.- essential oil
